# Effects of Mediterranean Diet and Physical Activity on Pulmonary Function: A Cross-Sectional Analysis in the ILERVAS Project

**DOI:** 10.3390/nu11020329

**Published:** 2019-02-03

**Authors:** Liliana Gutiérrez-Carrasquilla, Enric Sánchez, Marta Hernández, Dinora Polanco, Jordi Salas-Salvadó, Àngels Betriu, Anna Michela Gaeta, Paola Carmona, Francesc Purroy, Reinald Pamplona, Cristina Farràs, Carolina López-Cano, Elvira Fernández, Albert Lecube

**Affiliations:** 1 Endocrinology and Nutrition Department, University Hospital Arnau de Vilanova, Obesity, Diabetes and Metabolism (ODIM) research group, IRBLleida, University of Lleida, 25195 Lleida, Spain; liligutierrezc@gmail.com (L.G.-C.); esanchez@irblleida.cat (E.S.); martahernandezg@gmail.com (M.H.); karolopezc@gmail.com (C.L.-C.); 2 Respiratory Department, University Hospital Arnau de Vilanova-Santa María, Translational Research in Respiratory Medicine, IRBLleida, University of Lleida, 25198 Lleida, Spain; din4pa@hotmail.com (D.P.); annamichelagaeta@hotmail.it (A.M.G.); pcarmona@gss.scs.es (P.C.); efernandez@irblleida.cat (E.F.); 3 Centro de Investigación Biomédica en Red de Enfermedades Respiratorias (CIBERES), Instituto de Salud Carlos III (ISCIII), 28029 Madrid, Spain; 4 Human Nutrition Unit, Biochemistry and Biotechnology Department, Faculty of Medicine and Health Sciences, University Hospital of Sant Joan de Reus, IISPV, Rovira i Virgili University, 43201 Reus, Spain; jordi.salas@urv.cat; 5 Centro de Investigación Biomédica en Red de Fisiopatología de la Obesidad y la Nutrición (CIBEROBN), Instituto de Salud Carlos III (ISCIII), 28029 Madrid, Spain; 6 Unit for the Detection and Treatment of Atherothrombotic Diseases (UDETMA V&R), University Hospital Arnau de Vilanova, Vascular and Renal Translational Research Group, IRBLleida. University of Lleida, 25198 Lleida, Spain; angels.betriu.bars@gmail.com; 7 Stroke Unit. University Hospital Arnau de Vilanova, Clinical Neurosciences Group, IRBLleida. University of Lleida, 25198 Lleida, Spain; fpurroygarcia@gmail.com; 8 Experimental Medicine Department. IRBLleida, University of Lleida, 25198 Lleida, Spain; reinald.pamplona@mex.udl.cat; 9 Primary Health Care Unit, 25007 Lleida, Spain; cfarras.lleida.ics@gencat.cat; 10 Centro de Investigación Biomédica en Red de Diabetes y Enfermedades Metabólicas Asociadas (CIBERDEM), Instituto de Salud Carlos III (ISCIII), 28029 Madrid, Spain

**Keywords:** forced vital capacity, forced expiratory volume in the first second, lung function, Mediterranean diet, physical activity, questionnaire

## Abstract

A few studies showed that both adherence to Mediterranean diet (MedDiet) and physical activity practice have a positive impact on pulmonary function in subjects with lung disease. These associations are not well studied in subjects free from lung disease. In a cross-sectional study conducted in 3020 middle-aged subjects free of lung disease, adherence to the MedDiet using the Mediterranean Diet Adherence Screener, and physical activity practice using the International Physical Activity Questionnaire short form were recorded. Respiratory function was assessed using forced spirometry and the results were evaluated according to the Global initiative for Chronic Obstructive Lung Disease. Logistic regression models were used to analyze the associations between adherence to the MedDiet and physical activity practice with the presence of ventilatory defects. Participants with a high adherence to MedDiet, in comparison to those with low adherence, had both higher forced vital capacity (FVC; 100 (87–109) vs. 94 (82–105) % of predicted, *p* = 0.003) and forced expired volume in the first second (FEV1; 100 (89–112) vs. 93 (80–107) % of predicted, *p* < 0.001). According to their degree of physical activity, those subjects with a high adherence also had both higher FVC (100 (88–107) vs. 94 (83–105) % of predicted, *p* = 0.027) and FEV1 (100 (89–110) vs. 95 (84–108) % of predicted, *p* = 0.047) in comparison with those with low adherence. The multivariable logistic regression models showed a significant and independent association between both low adherence to MedDiet and low physical activity practice, and the presence of altered pulmonary patterns, with differences between men and women. However, no joint effect between adherence to MedDiet and physical activity practice on respiratory function values was observed. Low adherence to MedDiet and low physical activity practice were independently associated with pulmonary impairment. Therefore, the lung mechanics seem to benefit from heart-healthy lifestyle behaviors.

## 1. Introduction

The Mediterranean diet (MedDiet) is characterized by an abundant consumption of extra-virgin olive oil, fruits, vegetables, nuts, and legumes, a moderate consumption of fish and seafood, poultry, fermented dairy products, and red wine (with meals), and low intakes of sweetened beverages, red meat, and ready meals [1,2]. This traditional dietary pattern is of interest for health due to observations from the 1960s that populations bordering the Mediterranean Sea experienced lower mortality from cardiovascular diseases [3,4,5]. Subsequent observational studies expanded the benefits of the MedDiet pattern to other diseases such as obesity, metabolic syndrome, type 2 diabetes, cardiovascular diseases, certain types of cancer, and some neurodegenerative diseases [6,7,8,9,10,11]. The MedDiet can exert its effect through different pathophysiological mechanisms, such as decreasing inflammation, improving the lipid profile, and reducing blood pressure and insulin resistance, among others [12,13,14,15].

A relationship between MedDiet adherence and pulmonary function was also evidenced during the last decade. In this way, there is growing evidence about the beneficial effect of the MedDiet on lung function in patients with chronic obstructive pulmonary disease (COPD), asthma, cystic fibrosis. and smoking, suggesting that inflammatory modulation of the diet may play an important role [16,17,18,19]. However, no data on healthy populations without pulmonary disease are available. 

On the other hand, the potential advantageous role of physical activity on the respiratory muscles’ strength and function was reported in patients with cystic fibrosis, COPD, obesity, and healthy adults [20,21,22,23]. Nevertheless, the associative and synergistic impact of the MedDiet and physical activity on lung function was not previously evaluated. 

Therefore, the main objective of the present study was to evaluate the associations between adherence to the MedDiet and physical activity practice on pulmonary function in a large middle-aged population at low-to-moderate cardiovascular risk.

## 2. Materials and Methods

### 2.1. Design of the Study and Description of the Study Population

A total of 3020 subjects were enrolled between 2015 and 2017 from different primary healthcare centers in an ongoing study dealing with subclinical atherosclerosis in Lleida, Catalonia, Spain Ilerda Vascular (ILERVAS) project, ClinTrials.gov Identifier: NCT03228459) [24]. The inclusion criteria were as follows: women between the ages of 50 and 70 and men between the ages of 45 and 65 with the presence of at least one cardiovascular risk factor (such as dyslipidemia, hypertension, obesity, smoking, or having a first-degree relative with premature cardiovascular disease). The exclusion criteria were the presence of known pulmonary disease, prior medical history of cardiovascular disease, any type of diabetes mellitus, chronic kidney disease, active neoplasia, a life expectancy less than 18 months, and pregnancy. The Adult Treatment Panel III guidelines were followed to classify participants into high risk (clinical coronary heart disease or a clinical coronary heart disease risk equivalent, such as other clinical forms of atherosclerotic disease (peripheral arterial disease, abdominal aortic aneurysm, and symptomatic carotid artery disease) and diabetes), moderate risk (two or more cardiovascular risk factors), and low risk (0–1 risk factors) [25].Therefore, this is an initial cohort of asymptomatic population with a low-to-moderate cardiovascular risk that will be followed up until January 2025 to observe the onset of cardiovascular events [24,25].

### 2.2. Pulmonary Function Measurements

Forced spirometry was done using a portable ultrasonic spirometer (Datospir^©^, Sibelmed, Barcelona, Spain). Pulmonary function examinations were performed by trained and certified pulmonary experts in agreement with the American Thoracic Society and European Respiratory Society guidelines [26,27]. Subjects were required to achieve at least three reproducible maneuvers, and the outputs that produced the highest total of forced vital capacity (FVC) and forced expiratory volume in the first second (FEV1) were selected for the analysis. A bronchodilator test was not included in the evaluation of pulmonary function. The spirometric parameters were measured as a percentage of the predicted values, and included FVC, FEV1, and the relationship between them (FEV1/FVC). 

An anomalous FEV1 was defined as a value lower than 80% of that predicted. In addition, a “non-obstructive ventilatory defect” was well defined by an FVC <80% of the predicted value with an FEV1/FVC ratio ≥70%, with a flow-volume curve showing a convex pattern. Finally, an “obstructive ventilatory defect”, an unequal reduction of greatest airflow in relation to the maximal volume that can be displaced from the lung, was defined by an FEV1/FVC <70% according to the Global Initiative for Chronic Obstructive Lung Disease (GOLD) [27].

### 2.3. Adherence to Mediterranean Diet Assessment

To quantitatively estimate the adherence to the MedDiet, we used the validated 14-item Mediterranean Diet Adherence Screener (MEDAS) that was developed to rapidly control for compliance with the dietary intervention in the Prevención con Dieta Mediterránea (PREDIMED) trial [4,28]. Final scores ranged from 0 to 14 and categorized subjects according to their level of adherence to MedDiet: (i) high (score ≥ 11 points), (ii) moderate (7–10 points), and (iii) low (≤6 points) [28,29].

### 2.4. Physical Activity Level and Type Assessment 

All participants also participated in the short version of The International Physical Activity Questionnaire (IPAQ), a questionnaire developed for checking adults’ physical activity and inactivity [30]. The IPAQ short form inquires about three detailed types of activity undertaken in four domains: leisure time, domestic and gardening, work-related, and transport-related physical activity. The specific types of activity that were assessed were walking, and moderate-intensity and vigorous-intensity actions. The metabolic equivalent of task (MET), a multiple of the resting metabolic rate, was calculated and was expressed in METs per week. Following IPAQ guidelines, the participants were classified as engaged in vigorous physical activity, moderate physical activity, and low physical activity [30]. 

### 2.5. Covariate Assessment

The smoking habit (never, former, or current smoker) was documented. Smokers who stopped smoking ≥1 year prior to enrolment were considered former smokers. Body weight and height were measured without shoes and slight clothes, and body mass index (BMI) was calculated from weight (kg) divided by height (meters) squared. Blood pressure was measured in triplicate, after relaxing at 2-min intervals, and the mean of the last two values was calculated (Omron M6, Omron Healthcare Europe BV, Hoofddorp, The Netherlands). Dried capillary blood testing was carried out to obtain levels of total cholesterol (mg/dL) using the system Reflotron^®^ plus (Roche Diagnostics, Mannhein, Germany). 

### 2.6. Ethical Approval 

The protocol was approved by the Arnau de Vilanova University Hospital ethics committee (CEIC-1410). Additionally, the study was conducted according to the ethical guidelines of the Helsinki Declaration, and Spanish legislation regarding the protection of personal information was also followed. Informed consent was obtained from all individual participants included in the study.

### 2.7. Statistical Analysis

The normal distribution of the variables was evaluated using the Shapiro-Wilk test. Owing to its skewed distribution, quantitative information was described using number (percentage) or the median (interquartile range). Participants were classified into three groups according to adherence to the MedDiet (high, moderate, and low) and in three other categories according to the frequency of physical activity (vigorous, moderate, and low). Main clinical data across categories of both MedDiet and physical activity were compared using the Krustal-Wallis test (with a Bonferroni post hoc analysis for pairwise comparisons) for continuous variables. The Pearson’s chi-squared test was used for categorical data. In addition, the relationship between continuous variables was assessed using the Spearman correlation test.

Three multivariable logistic regression models for the presence of an abnormal FEV1 (FEV1 < 80% of predicted), as well as non-obstructive and obstructive ventilatory defects for the development cohort, were done for each gender including the following confounding factors: age, body mass index, and both the adherence to the MedDiet and physical activity. The calibration and the discrimination of the logistic model were evaluated using the Hosmer-Lemeshow test of fit and the area under the receiver operating characteristic (ROC) curve, respectively. 

We explored the joint associations of combining the adherence to the MedDiet (three categories) and physical activity practice (three categories) with FEV1 and the presence of ventilatory defects. Therefore, each participant was cross-allocated to one of the nine joint categories, and the low adherence to the MedDiet plus low physical activity practice was considered as the reference category. The interaction between the adherence to the MedDiet and physical activity practice in their associations with each outcome was examined by calculating the likelihood ratio test between the fully adjusted model and the same model including the interaction product.

All *p*-values were based on a two-sided test of statistical significance. Significance was accepted at the level of *p* < 0.050. All statistical analyses were completed using SSPS statistical package (IBM SPSS Statistics for Windows, Version 20.0., IBM Corp, Armonk, NY, USA).

## 3. Results 

**Figure 1:**
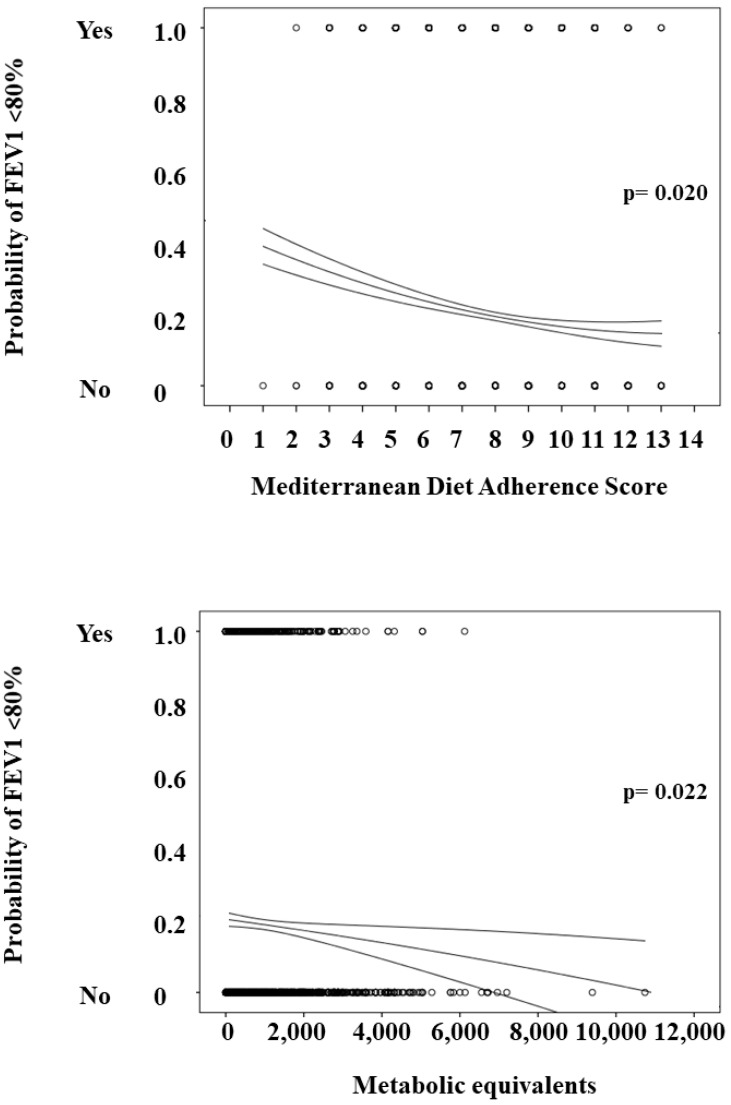
Plot displaying the presence of forced expired volume in the first second (FEV1) <80 according to the Mediterranean Diet Adherence Screener (MEDAS) score and physical activity measured as metabolic equivalents.

Adherence to the MedDiet was primarily moderate (80.1%) in our middle-aged population without known pulmonary disease. Main clinical data according to levels of adherence to the MedDiet are displayed in Table 1. Participants with high adherence to the MedDiet were older, showed a superior prevalence of women and non-smoker subjects, and expended more weekly energy in comparison to subjects with lower adherence. In addition, participants who scored ≥11 points in the MEDAS questionnaire also exhibited significantly higher values of FVC and FEV1 than subjects with lower values (FVC: 100 (87–109) vs. 94 (82–105) % of predicted, *p* = 0.003; FEV1: 100 (89–112) vs. 93 (80–107) % of predicted, *p* < 0.001), together with a lower prevalence of abnormal FEV1 and pulmonary patterns. Similar results were observed when participants with high and moderate adherence to the MedDiet were compared (Table 1). In addition, the total score of the MEDAS questionnaire was slightly and negatively correlated with both FVC and FEV1 value in the bivariate analysis (*r* = −0.046, *p* = 0.012 and *r* = −0.063, *p* = 0.001, respectively). As the MEDAS score results increased, the probability of anomalous FEV1 significantly declined (*p* = 0.020) (Figure 1).

Table 2 shows the clinical characteristics of the study population according to their physical activity measured by the IPAQ questionnaire. The physical activity was mostly low (62.2%) in our study population. Participants with vigorous physical activity were younger, mainly female, and with a lower body mass index in comparison to participants with low physical activity. However, their adherence to the MedDiet was similar between the three groups. Furthermore, when compared with participant with low physical activity, subjects with vigorous activity exhibited higher FVC (100 (88–107) vs. 94 (83–105) % of predicted, *p* = 0.027) and higher FEV1 (100 (89–110) vs. 95 (84–108) % of predicted, *p* = 0.047), together with lower prevalence of abnormal FEV1 or ventilatory defects (Table 2). In the bivariate analysis, the final score of the IPAQ questionnaire showed a negative correlation with FVC and FEV1 values (*r* = −0.102 and *r* = −0.073 respectively, *p* < 0.001 for both comparisons). Additionally, as the metabolic equivalents increased, the presence of abnormal FEV1 also decreased (*p* = 0.022) (Figure 1). 

The multivariable logistic regression models (Table 3 and Table 4) showed that, in women, low and moderate adherence to the MedDiet were significant and independently associated with the presence of abnormal FEV1 and a non-obstructive ventilatory pattern, respectively. However, in men, low adherence to the MedDiet only predicted the existence of an obstructive ventilatory defect.

Finally, no interaction between adherence to the MedDiet and physical activity with the presence of abnormal FEV1, non-obstructive ventilatory patterns, or obstructive ventilatory patterns were observed.

## 4. Discussion

The interventions to promote dietary lifestyle and physical activity changes for cardiovascular risk reduction are well established in adults [3,4,5,31,32]. This is the first study to analyze the associations between some lifestyle behaviors and respiratory function in a median-age population free from lung diseases but with the presence of at least one cardiovascular risk factor. Interestingly, a low adherence to the MedDiet was associated with impaired spirometric values and higher prevalence of abnormal lung function when compared to participants with high adherence to this dietary pattern. Similarly, vigorous physical activity was accompanied by better results in lung function than that observed in inactive subjects. However, in our population, with a low percentage of participants with high adherence to the MedDiet (7.4%) and vigorous physical activity (3.3%), the coexistence of both characteristics was not associated with better spirometric results. 

The MedDiet is a traditional dietary pattern that demonstrated valuable effects on health, quality of life, and longevity [1,2,33]. In fact, the MedDiet was shown to prevent cardiovascular events and premature total mortality owing its hypocholesterolemic, anti-inflammatory, and antioxidant properties [12,13,14,15,34]. In addition, a potential beneficial effect of the MedDiet on pulmonary function was previously evaluated in cross-sectional studies carried out in smokers and subjects with lung diseases, such as asthma, COPD, and cystic fibrosis [16,17,18,19]. Especially in asthma, the importance of the dietary pattern was highlighted in two systematic reviews and meta-analyses, concluding that adherence to the MedDiet may be effective in the prevention of asthma or wheeze in children; however, these associations are controversial in the case of adults [35,36]. Our study expands these results to a population without pulmonary disease, because, compared to those participants with high adherence to the MedDiet, those with low adherence had lower FVC and FEV1 values during spirometric maneuvers and had a higher prevalence of abnormal lung function patterns. 

The mechanisms to explain the cluster of pulmonary benefits associated with high adherence to the MedDiet are not yet fully understood. However, it was argued that the most suitable explanation could be related with its anti-inflammatory and antioxidant properties associated with the high content of carotenoids, polyphenols, polyunsaturated fatty acids, and antioxidants contained in the food groups defining the MedDiet [37,38,39,40,41]. In this way, in case of the Atherosclerosis Risk in Communities (ARIC) cohort, the European Community Respiratory Health Survey (ECRHS), and other studies, it was recognized that the combination and interaction of several nutrients may be more important than individual dietary components [39,41]. More recently, alterations in gut microbiome modulated by dietary intake were proposed as additional potential mechanisms modulating lung function [42].

Moreover, the effect of the MedDiet over a four-week period reduced serum advanced glycation end products (AGEs) compared to the effect of a Western diet rich in saturated fat [43]. Similarly, a three-month hypocaloric diet promoting the consumption of typically MedDiet food was able to decrease serum N(ε)-carboxymethyl-lysine in overweight/obese premenopausal women [44]. In this way, our group recently showed that skin AGE deposition was associated with lower spirometric values and higher prevalence of abnormal ventilatory patterns in the ILERVAS population, providing another mechanism explaining the association between diet and lung function [45]. 

On the other hand, strong evidence showed how physical activity practice may help patients with lung diseases, such as COPD, asthma, and cystic fibrosis, to improve better lung function and disease control [46,47,48]. Regarding healthy subjects, it is worth mentioning that higher cardiorespiratory fitness is associated with lesser decline in lung function across the lifespan [49]. Our results reinforce the protective effect of physical activity on lung function, showing how higher degrees of inactivity according to the IPAQ score are independent risk factors for pulmonary impairment assessed by FEV1 <80% and non-obstructive ventilatory pattern. 

Although, in our study, lung damage and respiratory abnormalities were of moderate magnitude and even subclinical, there could be a long-term deleterious impact. In this regard, after a 29-year follow-up of the Buffalo Health Study, FEV1 (% predicted) was demonstrated to be a long-term predictor for all-cause mortality, as well as ischemic heart disease [50]. Similarly, in another 26-year follow-up prospective study cohort including 1623 apparently healthy males aged 40–59 years, the relative risk for all-cause mortality, cardiovascular death, and respiratory death increased by 10%, 7%, and 34% for each 10% of reduction in FEV1 [51]. These results were obtained after adjusting for smoking, age, systolic blood pressure, BMI, serum cholesterol, and physical fitness [51].

Our results showed a sexual dimorphism regarding the impact of the MedDiet on pulmonary function. It was previously suggested that lung function may be influenced by sex-linked biological differences [52]. Dominelli et al. described that central airways in healthy women are significantly smaller than men, by approximately 30% [53]. Similarly, female smokers appeared to experience an accelerated decline in FEV1 compared with male smokers [54]. Additionally, women are usually exposed to different respiratory occupational risk factors than men [55]. Our data, with a different impact of the MedDiet on pulmonary function according to sex, reinforce the relevance of this variable when evaluating lung function.

The finding in our study of no interaction between adherence to the MedDiet and physical activity with pulmonary function deserves a comment, as their synergic effect was observed in other clinical situations. In this way, in people with impaired glucose tolerance, diet plus physical activity decreases the risk of developing type 2 diabetes compared to standard treatment, without firm evidence of the effect of both components alone [56]. Similarly, a combined diet-plus-exercise program for a minimum of six months provided greater weight loss than a diet-only program among obese or overweight adults in eighteen randomized controlled trials [57]. The effectiveness of exercise mode (aerobic training, resistance training, or combined) in influencing lung function also needs to be considered, as different effects were reported in improving functional status of dieting obese older adults [58]. In our study, the sample of participants with high adherence to the MedDiet was low, similar to the percentage of subjects who undertook vigorous physical activity (7.4% and 3.3%, respectively), preventing us from achieving sufficient statistical power to analyze the joint associations of combining adherence to the MedDiet and physical activity.

There are some potential limitations that should be considered in evaluating the results of our study. Firstly, the cross-sectional characteristic of the study limits us from establishing a causal relationship of the reported associations. Secondly, a post-bronchodilator spirometry to confirm the obstructive pattern was not conducted in our population. This test reduces the overall prevalence of COPD by approximately 33% in community-based health check-ups [59]. Thirdly, results from this study cannot be extrapolated to other populations, since our population comprised Spanish middle-aged individuals at low-to-moderate cardiovascular risk. Finally, no data about the total calorie intake are available from participants in our study. Moreover, the present study also has some strengths: the large study sample, the validated tools used, and the adjustment of the models for a large number of potential confounders.

To sum up, the present study provides initial clinical evidence about the independent and deleterious effect of both low adherence to the MedDiet and low physical activity practice on lung function in subjects without known pulmonary disease. More prospective and clinical trial interventions are warranted in the future to confirm the effectiveness of a healthy diet and physical activity in preventing respiratory disorders.

## Figures and Tables

**Table 1:** Main clinical data, comorbidities, and indicators of pulmonary function of the study population according to adherence to Mediterranean diet.

Variables	Low Adherence	Moderate Adherence	High Adherence	*p* *	*p* **
*n* (%)	376 (12,4)	2420 (80.1)	224 (7.4)	-	-
Women, *n* (%)	154 (41.0)	1400 (57.9)	136 (60.7)	<0.001	0.436
Age (years)	55 (51–61)	58 (53–64)	59 (54–64)	<0.001	1.000
Hypertension, *n* (%)	135 (35.9)	100 (41.3)	88 (39.3)	0.432	0.571
Systolic blood pressure (mmHg)	130 (120–142)	131 (120–143)	129 (118–141)	0.429	0.429
Diastolic blood pressure (mmHg)	82 (76–88)	82 (75–88)	80 (74–88)	0.143	0.143
Dyslipidemia, *n* (%)	186 (49.5)	1284 (53.1)	127 (56.7)	0.092	0.327
Total cholesterol (mg/dL)	202 (178–228)	204 (181–229)	208 (184–235)	0.147	0.147
Obesity, *n* (%)	90 (23.9)	701 (29.0)	52 (23.2)	0.921	0.075
BMI (kg/m^2^)	28.4 (25.6–31.2)	28.5 (25.7–31.6)	27.6 (25.3–30.4)	0.277	0.015
Current smoker, *n* (%)	147 (39.1)	559 (23.1)	40 (17.9)	<0.001	0.026
Total METs per week	480 (198–1188)	720 (240–1272)	975 (396–1386)	<0.001	0.012
FVC (% predicted)	94 (82–105)	95 (84–107)	100 (87–109)	0.003	0.020
FEV1 (% predicted)	93 (80–107)	97 (84–108)	100 (89–112)	<0.001	0.009
FEV1/FVC	78 (73–83)	78 (74–82)	79 (75–83)	0.273	0.273
FEV1 < 80% predicted, *n* (%)	91 (24.3)	402 (16.7)	31 (13.8)	0.002	0.300
Non-obstructive ventilatory defect ^i^, *n* (%)	61 (16.2)	334 (13.8)	21 (9.4)	0.019	0.065
Obstructive ventilatory defect ^i^, *n* (%)	55 (14.6)	298 (12.3)	16 (7.1)	0.006	0.023

* Low vs. high adherence to Mediterranean diet; ** moderate vs. high adherence to Mediterranean diet. Data are expressed as medians (interquartile range) or *n* (percentage). BMI: body mass index; METs: metabolic equivalent of task; FVC: forced vital capacity; FEV1: forced expired volume in the first second; ^i^ according to the Global Initiative for Chronic Obstructive Lung Disease.

**Table 2:** Main clinical data, comorbidities, and pulmonary function of the study population according to physical activity practice.

Variables	Low Physical Activity	Moderate Physical Activity	Vigorous Physical Activity	*p* *	*p* **
*n* (%)	1880 (62.2)	1039 (34.4)	101 (3.3)	-	-
Women, *n* (%)	975 (51.9)	679 (65.4)	36 (35.6)	0.001	<0.001
Age (years)	57 (53–63)	59 (54–64)	54 (50–61)	0.004	<0.001
Hypertension, *n* (%)	747 (39.8)	440 (42.3)	35 (34.7)	0.347	0.140
Systolic blood pressure (mmHg)	131 (120–143)	131 (120–143)	126 (118–140)	0.274	0.274
Diastolic blood pressure (mmHg)	82 (75–88)	81 (75–88)	78 (73–88)	0.157	0.157
Dyslipidemia, n (%)	987 (52.5)	560 (53.9)	49 (48.5)	0.474	0.347
Total cholesterol (mg/dL)	204 (180–229)	204 (184–230)	195 (170–218)	0.025	0.007
Obesity, *n* (%)	531 (28.3)	295 (28.4)	16 (15.8)	0.006	0.007
BMI (kg/m^2^)	28.6 (25.8–31.2)	28.3 (25.4–31.6)	27.5 (24.5–30.4)	0.005	0.025
Current smoker, *n* (%)	472 (25.1)	241 (23.2)	32 (31.7)	0.122	0.022
MedDiet score	8 [7,8,9]	8 (7–10)	8 (7–9)	1.000	1.000
FVC (% predicted)	94 (83–105)	97 (85–108)	100 (88–107)	0.027	0.703
FEV1 (% predicted)	95 (84–108)	98 (84–111)	100 (89–110)	0.047	0.588
FEV1/FVC	79 (74–83)	79 (73–82)	79 (74–82)	1.000	1.000
FEV1 < 80% predicted, *n* (%)	330 (17.7)	185 (17.9)	9 (8.9)	0.021	0.026
Non-obstructive ventilatory defect ^i^, *n* (%)	281 (15.0)	128 (12.3)	7 (6.9)	0.029	0.145
Obstructive ventilatory defect ^i^, *n* (%)	213 (11.3)	141 (13.6)	15 (14.9)	0.265	0.761

* Low vs. high adherence to physical activity; ** moderate vs. high adherence to physical activity. Data are expressed as medians (interquartile range) or *n* (percentage). BMI: body mass index; MedDiet score: total score from the Mediterranean Diet Adherence Screener (MEDAS); FVC: forced vital capacity; FEV1: forced expired volume in the first second; ^i^ according to the Global Initiative for Chronic Obstructive Lung Disease.

**Table 3:** A multivariable logistic regression model for the presence of FEV1 <80% predicted and both non-obstructive and obstructive ventilatory defects in women.

FEV1 < 80%		OR (95% CIs) *	*p*
Age (years)		0.99 (0.98–1.02)	0.893
BMI (kg/m^2^)		1.02 (1.00–1.05)	0.123
Adherence to Mediterranean diet	High	Reference	
	Moderate	1.27 (0.73–2.22)	0.404
	Low	2.07 (1.06–4.06)	0.033
Physical activity practice	Vigorous	Reference	
	Moderate	1.42 (1.49–4.12)	0.516
	Low	1.22 (0.42–3.52)	0.711
Hosmer–Lemeshow test of fit			0.713
Area under the ROC curve		0.54 (0.50–0.59)	0.028
**Non-obstructive ventilatory defect**			
Age (years)		1.02 (0.99–1.05)	0.084
BMI (kg/m^2^)		1.06 (1.03–1.09)	<0.001
Adherence to Mediterranean diet	High	Reference	
	Moderate	2.21 (1.01–4.83)	0.047
	Low	2.42 (0.97–6.05)	0.058
Physical activity practice	Vigorous	Reference	
	Moderate	3.83 (0.51–28.7)	0.191
	Low	4.41 (0.59–32.8)	0.147
Hosmer–Lemeshow test of fit			0.026
Area under the ROC curve		0.59 (0.55–0.63)	<0.001
**Obstructive ventilatory defect**			
Age (years)		1.06 (1.03–1.09)	<0.001
BMI (kg/m^2^)		0.95 (0.92–0.98)	0.001
Adherence to Mediterranean diet	Vigorous	Reference	
	Moderate	1.55 (0.84–2.88)	0.164
	Low	1.99 (0.93–4.26)	0.077
Physical activity practice	High	Reference	
	Moderate	0.76 (0.30–1.91)	0.559
	Low	0.68 (0.27–1.69)	0.402
Hosmer–Lemeshow test of fit			0.160
Area under the ROC curve		0.62 (0.57–0.66)	<0.001

* Independent variables included in the analysis were age, gender, body mass index, adherence to Mediterranean diet, and physical activity practice. ROC: receiver operating characteristic; OR: odds ratio; CI: confidence interval.

**Table 4:** A multivariable logistic regression model for presence of FEV1 <80% predicted and both non-obstructive and obstructive ventilatory defects in men.

FEV1 < 80%		OR (95% CIs) *	*p*
Age (years)		1.04 (1.02–1.06)	<0.001
BMI (kg/m^2^)		1.04 (1.00–1.07)	0.032
Adherence to Mediterranean diet	High	Reference	
	Moderate	1.17 (0.66–2.07)	0.587
	Low	1.75 (0.94–3.27)	0.078
Physical activity practice	Vigorous	Reference	
	Moderate	3.10 (1.20–8.03)	0.020
	Low	2.95 (1.16–7.49)	0.023
Hosmer–Lemeshow test of fit			0.273
Area under the ROC curve		0.60 (0.56–0.63)	<0.001
**Non-obstructive ventilatory defect**			
Age (years)		1.05 (1.02–1.08)	<0.001
BMI (kg/m^2^)		1.08 (1.04–1.12)	<0.001
Adherence to Mediterranean diet	High	Reference	
	Moderate	1.06 (0.58–1.94)	0.851
	Low	1.32 (0.68–2.59)	0.413
Physical activity practice	Vigorous	Reference	
	Moderate	1.56 (0.64–3.82)	0.331
	Low	1.85 (0.78–4.39)	0.165
Hosmer–Lemeshow test of fit			0.353
Area under the ROC curve		0.59 (0.55–0.63)	<0.001
**Obstructive ventilatory defect**			
Age (years)		1.04 (1.01–1.07)	0.006
BMI (kg/m^2^)		0.97 (0.93–1.01)	0.184
Adherence to Mediterranean diet	Vigorous	Reference	
	Moderate	3.15 (1.13–8.76)	0.028
	Low	4.14 (1.42–12.1)	0.009
Physical activity practice	High	Reference	
	Moderate	0.99 (0.46–2.15)	0.983
	Low	0.74 (0.35–1.56)	0.434
Hosmer–Lemeshow test of fit			0.210
Area under the ROC curve		0.60 (0.56–0.65)	<0.001

* Independent variables included in the analysis were age, gender, body mass index, adherence to Mediterranean diet, and physical activity practice. ROC: receiver operating characteristic; OR: odds ratio; CI: confidence interval.

## Supplementary Materials

The following are available online at http://www.mdpi.com/2072-6643/11/2/329/s1, Author list and affiliations of ILERVAS project investigators.
