# Supplementary material for: Effects of Mediterranean Diet and Physical Activity on Pulmonary Function: A Cross-Sectional Analysis in the ILERVAS Project
Source: Nutrients. 2019 Feb 3;11(2):329. doi: 10.3390/nu11020329 (PMC6413220; doi:10.3390/nu11020329)
Supplement: Supplementary file 1 [file nutrients-11-00329-s001.pdf]

Supplementary Material:

Author list and affiliations of ILERVAS project investigators.

Manuel Sánchez-de-la-Torre [1,2], Gerard Torres [1,2], Guillermo Suárez [1], Ferrán Barbé [1,2], Ferran Rius [3], Marcelino Bermúdez-López [4], Montse Martínez-Alonso [4], José Manuel Valdivielso [4], Laura Colàs-Campàs [5], Ikram Benabdelhak [5], Manuel Portero-Otin [6], Mariona Jové [6], Marta Ortega [7], Eva Miquel [7].

[1] Respiratory Department, University Hospital Arnau de Vilanova-Santa María, Translational Research in Respiratory Medicine, IRBLleida, University of Lleida, 25198 Lleida, Spain; sanchezdelatorre@gmail.com; gtorres@gss.scs.es; gsuarez@gmail.com; febarbe.lleida.ics@gencat.cat.
[2] Centro de Investigación Biomédica en Red de Enfermedades Respiratorias (CIBERES), Instituto de Salud Carlos III (ISCIII), 28029 Madrid, Spain; sanchezdelatorre@gmail.com; gtorres@gss.scs.es; febarbe.lleida.ics@gencat.cat.
[3] Endocrinology and Nutrition Department, University Hospital Arnau de Vilanova, Obesity, Diabetes and Metabolism (ODIM) research group, IRBLleida, University of Lleida, 25198 Lleida, Spain; friusriu@gmail.com.
[4] Unit for the Detection and Treatment of Atherothrombotic Diseases (UDETMA V&R), University Hospital Arnau de Vilanova, Vascular and Renal Translational Research Group, IRBLleida, University of Lleida, 25198 Lleida, Spain; mbermudez@irblleida.cat; montserrat.martinez@cmb.udl.cat; valdivielso@medicina.udl.cat.
[5] Stroke Unit, University Hospital Arnau de Vilanova, Clinical Neurosciences Group, IRBLleida, University of Lleida, 25198 Lleida, Spain; laura.colas.campas@gmail.com; ikram4@hotmail.com.
[6] Experimental Medicine Department. IRBLleida, University of Lleida, 25198 Lleida, Spain; manuel.portero@mex.udl.cat; mariona.jove@udl.cat.
[7] Primary Health Care Unit, 25007 Lleida, Spain; mortega.lleida.ics@gencat.cat; emiquel.lleida.ics@gencat.cat.
